# Slow nocturnal body cooling during sleep increases interbeat intervals and is tightly coupled to high‐frequency heart rate variability in healthy men

**DOI:** 10.14814/phy2.70478

**Published:** 2025-08-07

**Authors:** Kurt Kräuchi, Martin Glos, Ingo Fietze, Thomas Penzel, Matteo Mason, Sebastian Herberger

**Affiliations:** ^1^ Psychiatric University Clinics University of Basel Basel Switzerland; ^2^ Interdisciplinary Center of Sleep Medicine Charité – Universitätsmedizin Berlin Berlin Germany; ^3^ Technogel Italia S.R.L. Vicenza Italy

**Keywords:** body cooling, body temperature, cooling mattress, heart rate‐variability, interbeat interval, sleep stages

## Abstract

During nocturnal sleep, core body temperature (CBT) decreases and cardiac inter‐beat intervals (IBI) increase. This study used a non‐disturbing cooling intervention during sleep to investigate additional effects of CBT reduction on IBI and heart rate variability. Sleep on a high heat‐capacity cooling gel mattress (HM) was compared to a low heat‐capacity mattress (LM) in 32 healthy males (age 46 ± 4 years). Electroencephalography, electrocardiography, CBT, skin, mattress, and air temperatures were recorded under constant conditions. Power in the low‐ (LF) and high‐frequency (HF) bands and the LF/HF ratio were calculated from one‐minute IBI intervals. Sleep on HM led to greater CBT decline and showed increased IBI and HF in all sleep stages. Mediation analyses indicate HF is not directly influenced by CBT, but HF changes follow IBI, which mediates CBT effects on HF. HF and LF differ between sleep stages: HF is lower during rapid eye movement (REM) and higher during non‐REM, independent of CBT or IBI. In sum, sleep on a cooling mattress decreases CBT, which increases IBI and HF, independent of sleep stages. Cooling‐induced HF changes are mediated by IBI. In contrast, HF and LF vary with sleep stages, reflecting autonomic activity throughout sleep cycles, independent of thermophysiological changes.

## 1 INTRODUCTION

It has long been recognized that, in addition to following their inherent circadian rhythms, core body temperature (CBT), heart rate (HR) and cardiac output are reduced during sleep compared to wakefulness (Krauchi & Deboer, 2010). These sleep‐induced processes evolve at a rather slow pace (Krauchi, 2007); however, there are additional rapid changes that occur along the non‐Rapid Eye Movement sleep (REM) – REM sleep (NREM‐REM) cycle, for example, for HR and heart rate variability (HRV) measures (Tobaldini et al., 2013). During NREM, the sympatho‐vagal balance shifts towards parasympathetic modulation, while during REM, the balance shifts towards sympathetic modulation (Tobaldini et al., 2013). HRV is often used as a measure for assessing the balance of the autonomic nervous system. The high‐frequency (HF, 0.15–0.4 Hz) component is assumed to indicate parasympathetic activity, while the low‐frequency (LF, 0.04–0.15 Hz) component may reflect combined sympathetic and parasympathetic activity (de Geus et al., 2019). Respiration also is subject to systematic modulation during sleep, becoming deeper and more regular with deeper sleep, and shallower and more frequent during REM sleep (Cabiddu et al., 2012; Penzel et al., 2016). The so‐called respiratory sinus arrhythmia is characterized by rhythmic fluctuations in HR that align with the breathing cycle. It plays a vital role in HRV, and spectral analysis typically reveals an HF component centered around the respiratory frequency (Penttila et al., 2001). The observed increase in the power of HF components within the heart period (inter‐beat‐interval), IBI, and respiratory signals, together with their heightened coherence, supports the notion of an enhanced parasympathetic drive during NREM (Penzel et al., 2016). The main question of our paper is: How are the thermoregulatory, cardiovascular, and sleep regulatory systems interwoven under non‐disturbing conditions? In contrast to small mammals, in which brain temperature and CBT decrease during NREM (including slow wave sleep, SWS = N3) and increase during REM (Alföldi et al., 1990), humans do not exhibit significant alterations in CBT and skin temperatures with respect to NREM‐REM cycles (Kräuchi & Deboer, 2013). Instead, as mentioned above, CBT follows a slow decline after lights off by a maximum of 0.3°C about in the middle of a night's sleep, which was independent of the NREM‐REM cyclicity (Krauchi, 2007). Previous works have shown a strong correlation of reductions in CBT to increases in IBI. To determine the interrelation of CBT, HR, and HRV during sleep, specific challenges to the thermoregulatory, cardiovascular, and sleep regulatory systems are required that do not disturb sleep. Thermal intervention during sleep is challenging because any temperature changes beyond the tolerable range can disturb sleep, especially cold environments (Haskell et al., 1981). However, such cooling experiments without disturbing sleep are needed to determine how body heat loss leading to reduced CBT, proximal back skin temperature (PBT) and HR, and how these changes are interrelated to changes in sleep. Successful examples of thermal intervention to affect sleep in the past have used changes in ambient temperature and humidity, bedding and bedclothes, or mattress type, among others (reviewed in e.g., Herberger et al., 2024). In recent years, we have shown how a high heat capacity (HM) mattress, in comparison to a low heat capacity mattress (LM), increases conductive body heat loss at a slow rate, reduces nocturnal CBT and HR, and selectively increases N3, without disturbing sleep (Chiba et al., 2018; Herberger et al., 2020; Kräuchi et al., 2018; Reid et al., 2021).

The aim of the present study was to examine whether and how a non‐disturbing, gentle reduction in CBT during nighttime sleep affects HR, as well as the HF and LF components of HRV, in relation to the nocturnal time course and sleep stages in middle‐aged, healthy men.

## 2 MATERIALS AND METHODS

### 2.1 Study population

Participants were recruited via small public notices and an online advertisement on the Charité intranet. Thirty‐two healthy male subjects (age: 46 ± 4 years, body mass index of 25.2 ± 1.8 kg/m^2^; mean ± SD) were enrolled in the study. Approval for the study was obtained from the local Ethics Committee (application number: EA1/316/15 of the University Hospital, Charité, Berlin), adhering to the principles outlined in the declaration of Helsinki and written consent was given from all participants. All subjects were confirmed as normal sleepers with no signs of sleep disturbances. Exclusion criteria included acute or chronic illness, use of hypnotic agents or other medications affecting sleep–wake regulation, known sleep disorders, chronic drug or alcohol abuse or dependence, or participation in other clinical‐pharmacological trials within 4 weeks prior to the examination. For further details see Herberger et al. (2020).

### 2.2 Study design

The study was structured as a randomized, double‐blinded, crossover trial to compare the high heat‐capacity mattress (HM) with a standard low heat‐capacity mattress (LM). This comparison was conducted through polysomnography (PSG), CBT, skin, mattress, and ambient air temperature measurements performed in parallel under constant laboratory conditions. Ambient indoor room temperature was recorded using an iButton sensor (Thermochron iButtons DS1922L, Maxim, Dallas) placed on the bedside table. Room temperature was maintained at a stable 20.5°C using air conditioning, with relative humidity kept at approximately 50%. The subjects wore the laboratory standard cotton nightclothes and were covered by a cotton sheet during the lights‐off phase. Each subject was randomly assigned to undergo one measurement night on HM, followed by a 1‐week washout period, and then another measurement night on LM, or vice versa.

### 2.3 Mattress properties

The HM comprises a foam core coated with a polyurethane high‐heat capacity material (Technogel, Italia S.R.L., Vicenza, Italy), while the LM is composed of 100% foam material. Both mattresses shared identical dimensions of 90*200*25 cm. Variations in thermal properties stemmed from differences in material density within the top 2 cm layer, where higher material density resulted in increased heat capacity within the studied temperature range of 23°C–35°C (HM: 47 kJ/°C; LM: 5.4 kJ/°C for the mattress surface top 2 cm). To mitigate potential confounding effects, identical bed covers (bi‐elastic non‐quilted textile, 600 g/m^2^) and pillows were utilized. Contact address for further information regarding the mattresses: Technogel, Italia S.R.L., Vicenza, Italy.

### 2.4 Temperature measurements

CBT was monitored using an ingestible telemetric capsule sensor (VitalSense Core Temperature Capsule, Hidalgo Ltd., Cambridge, UK) with a resolution of 0.01°C and a sampling rate of 4 per minute. Additionally, CBT, skin, ambient room, and mattress temperatures were recorded using wireless temperature sensors (Thermochron iButtons DS1922L, Maxim, Dallas) with a resolution of 0.0625°C and a sampling rate of 1 per minute. The iButtons were attached to the skin and mattresses using air‐permeable surgical tape (Fixomull; Beiersdorf, Hamburg, Germany).

To assess conductive heat loss from the body core to the mattress, the mean of 3 proximal back skin temperatures (PBT) and the mean of 5 mattress surface temperatures were calculated. It is worth noting that none of the subjects identified as “stomach sleepers” according to their reports. For further methodological details see (Herberger et al., 2020).

### 2.5 PSG recordings including ECG, and sleep scoring

Subjects underwent PSG recordings using the EMBLA N7000 system (Embla systems, Broomfield, CO, USA) according to American Academy of Sleep Medicine (AASM) guidelines (Berry et al., 2012). Continuous polysomnographic recordings were performed by EEG, EMG chin, and EOG. ECG was derived from precordial chest leads, body position from the PSG system, and blood oxygen saturation from the digital probe. All signals were sampled and stored at 512 Hz, with 16‐bit AD conversion. For further methodological details see (Glos et al., 2014; Penzel et al., 2016; Herberger et al., 2020). PSG recordings were evaluated and scored by a certified sleep technician according to the AASM criteria (Berry et al., 2012) blinded to the type of mattress used during PSG. This person had no knowledge about other variables, for example, of subject's mattress temperature. Resulting variables of sleep scoring were absolute time of sleep stages N1, N2, N3 (= NREM), and REM, as well as wake time (WAKE).

### 2.6 Heart rate data processing

The method used for HRV analysis has been extensively described in previous publications (Glos et al., 2014; Penzel et al., 2016). Briefly: the ECG signal underwent offline filtering using a Butterworth bandpass (0.3–70 Hz) and RR intervals were extracted using an R‐peak detection algorithm. Artifacts (e.g., noise, body movements) were identified and removed using a semi‐automatic algorithm. To assign HR and HRV indices to specific sleep stages during both the PSG baseline and PSG recovery, we conducted calculations in consecutive 1‐minute intervals across the entire recording period.

Based on a 12‐pole AR spectral analysis, we likewise calculated the low‐frequency spectral band (LF, 0.04–0.15 Hz) – which is modulated by both the sympathetic as well as by the vagal activity, the high‐frequency spectral band (HF, 0.15–0.4 Hz) – a marker of vagal activity. All HRV data analysis was performed according to the Task Force on HRV Standards (Malik & Task Force of the European Society of Cardiology and the North American Society of Pacing and Electrophysiology, 1996) using the software Somnologica Science 3.3.1 (Embla systems, Broomfield, CO, USA).

### 2.7 Data analysis

For statistical time‐course analyses, temperatures and sleep stages were aggregated into 10 min time segments. Previous studies have shown that both absolute and ln‐transformed data show comparable reliability, with slightly higher values for ln‐transformed data in the frequency‐domain metrics (e.g (Besson et al., 2025)). According to these findings, we present HF and LF as ln‐transformed data. Differences in sleep stages and temperatures between the HM and LM were examined temporally across the night in 10 min intervals. ANOVA with two repeated factors TIME (time course, 48*10 min) and MAT (mattress, HM vs. LM), and subjects (as random factor) was performed using the package “lmer” in R (Pinheiro et al., 2021). Post‐hoc comparisons were calculated with the R package “emmeans” using the false discovery rate for alpha correction of multiple post hoc comparisons (FDR, Benjamini–Hochberg procedure; threshold for significance *p* < 0.05) (Benjamini et al., 2001). Similar findings occurred with ln‐transformed or untransformed data.

The effect sizes of ANOVA‐derived effects are expressed as eta squares (*η* ^2^) calculated using the R package “rstatix” (small *η* ^2^ = 0.01; medium *η* ^2^ = 0.06; large *η* ^2^ = 0.14) (Cohen, 1988). R‐squares (*r* ^2^) were used as the effect size measure for linear regression analyses (small effect size, *r* ^2^ = 0.01; medium effect size, *r* ^2^ = 0.09; large effect size, *r* ^2^ = 0.25), while Cohen's *d* was employed for assessing the effect size of group mean differences (small effect size, *d* = 0.2; medium effect size, *d* = 0.5; large effect size, *d* = 0.8) (Cohen, 1988).

Mediation analyses were performed using R package “mediation”. The confidence intervals were estimated based on the nonparametric bootstrap method with 500 simulations. Group data are presented as mean ± SD, or as the upper and lower thresholds of the 95%‐confidence intervals. To assess potential multicollinearity among predictors, variance inflation factors (VIFs) were calculated using the R package “car”.

## 3 RESULTS

**FIGURES 1 and 2:**
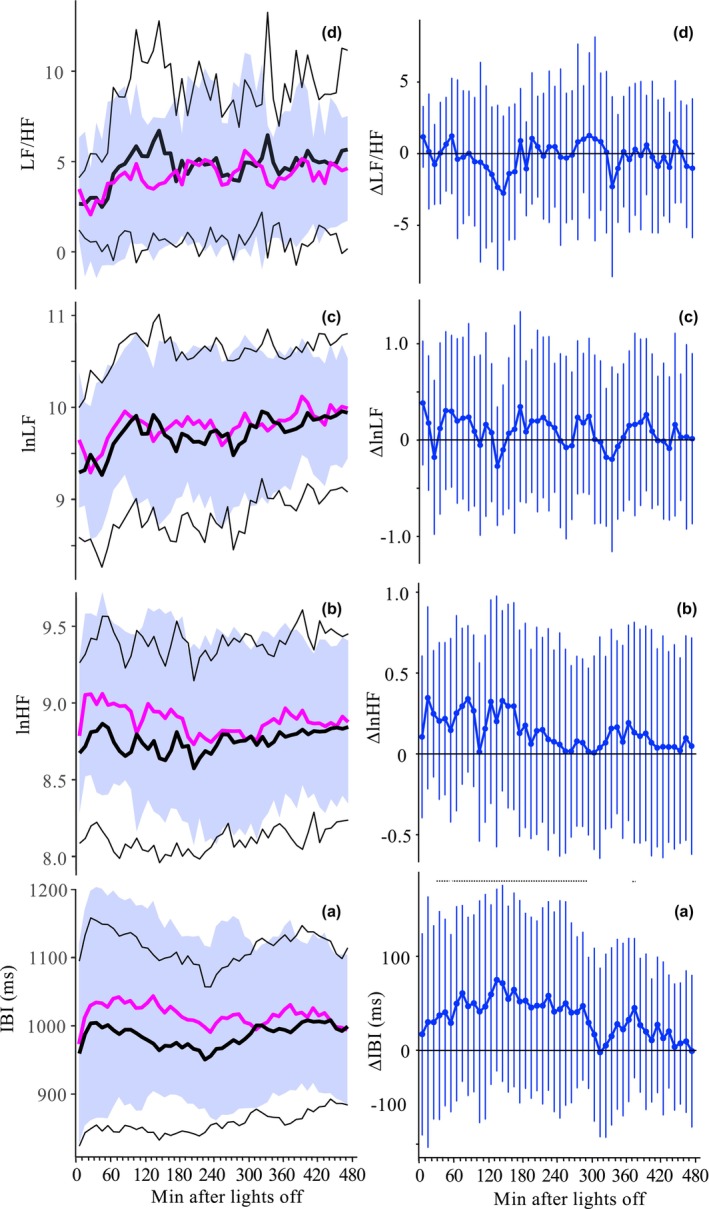
From bottom to top, the nocturnal time courses of HM and LM are shown for IBI, lnHF, lnLF and LF/HF (Fig.1, left panel, a–d) and for the differences between HM (blue) and LM (black) (Fig.2, right panel, a–d), [mean (thick lines) ± SD (thin lines, ribbon), *N* = 31, 48 × 10 min interval]. Black 0‐lines indicate no difference between HM and LM. For ∆IBI significant different values between HM and LM are indicated by a black dotted horizontal line (FDR, *p* < 0.05, a). IBI, interbeat interval; lnHF, natural logarithm of the power in the high frequency band (0.15–0.4 Hz), lnLF, natural logarithm of the power in the low frequency band (0.05–0.15 Hz); LF/HF, coefficient of power in the low frequency band/power in the high frequency band. LnHF and IBI exhibit similar time courses in HM, LM, and similar HM–LM differences.

**FIGURE 3:**
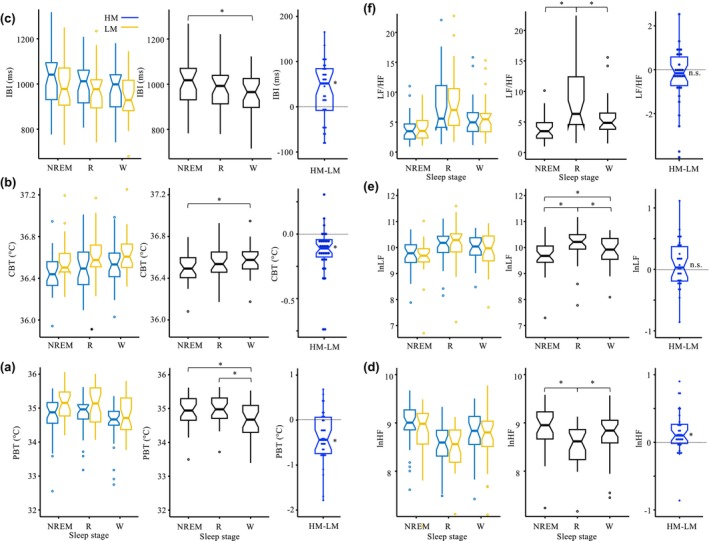
Visualization of the effects of sleep stages. Notched box‐and‐whisker plots illustrate the distributions of variables (a–f) under both mattress conditions (HM in blue; LM in orange) across three sleep stages (NREM, REM, WAKE) in three panels: By sleep stage (left), pooled across mattress conditions (middle in black), and as HM–LM differences over the 8‐h nocturnal period (right in blue). Each box represents the interquartile range (IQR), the line within the box indicates the median, and the notches represent approximate 95% confidence intervals around the medians. Whiskers extend to 1.5 * IQR, and outliers are shown as individual points. Statistical comparisons of HM–LM differences across sleep stages for all variables revealed no significant effects (all MAT*STAGE interactions *p* > 0.16; left panel). Comparisons between sleep stages (with HM and LM data pooled for each variable) showed significant differences for all variables (all main effects STAGE, *p* < 0.05; middle panel); significant post‐hoc differences between sleep stages are indicated by asterisks above the brackets (FDR‐adjusted *p* < 0.05). In the right panel, asterisks indicate significant differences in HM–LM values over the 8‐hour nocturnal period (main effect MAT, *p* < 0.05). For statistical tests see Table S2; a similar statistical outcome was found with non‐parametric tests (Freedman– and Wilcoxon–tests). For all variables, mattress‐induced effects are not significantly modulated by sleep stage. LnHf and IBI show distinct distributions across sleep stages, indicating that the relationship between these variables differs across sleep stages.

**FIGURE 4:**
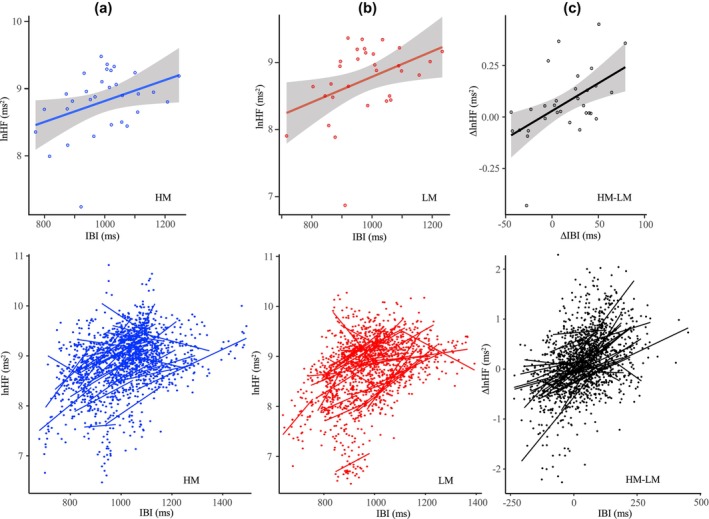
Visualization of the effects of IBI, MAT, and TIME on lnHF. Upper graph: Linear regression of the individual nocturnal means for IBI versus lnHF (*N* = 31 subjects) in condition MAT (HM, panel a vs. LM, panel b) and HM‐LM (panel c). Lower graphs: Visualization of the results of the linear mixed effects models (random intercept and slope model). Dots represent 48 × 10 min means per subject of IBI versus lnHF. The lines represent the individual linear fits for the 48 data pairs/subjects (factor TIME) in condition MAT (HM vs. LM) and HM‐LM. Both inter‐ and intra‐individual correlations of IBI with lnHF showed a significant linear association.

**FIGURE 5:**
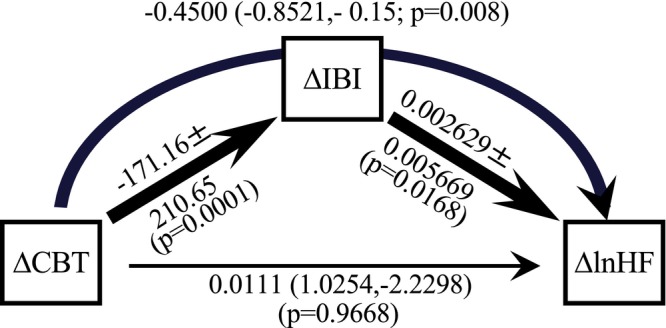
Visualization of the mediation analysis ∆CBT, ∆IBI, and ∆lnHF. Results of the mediation analysis of the mattress induced effects are summarized as a path diagram of the nocturnal mean values for the variables ∆CBT, ∆IBI and ∆lnHF. Mean path coefficients are presented beside the path arrows by SD or in brackets by the upper and lower thresholds of the 95%‐confidence intervals. Significant paths are indicated by thick arrows (*p* < 0.05), the thin line represents a non‐significant path (*N* = 31 subjects). CBT, core body temperature; IBI, inter beat interval; lnHF, natural logarithm of the power in the high frequency band (0.15–0.4 Hz). Bivariate correlation *r*‐values: ∆CBT versus ∆IBI = −0.637, *p* = 0.0001; ∆CBT versus ∆lnHF = −0.329; *p* = 0.0707, ∆IBI versus ∆lnHF = 0.5249, *p* = 0.0024. All ‘variance inflation factors’ (VIFs) were below 2 (∆CBT = 1.98, ∆IBI = 1.75, ∆CBT*∆IBI = 1.56), indicating no problematic multicollinearity among predictors. Cooling mattress induced effects on lnHF are significantly mediated by ∆IBI (indirect path, curved arrow).

Analyses of the collected data can be grouped into three physiological fields: sleep, body temperatures, and cardiophysiology. Based on the fact that we randomized the order of the mattress types across the study days, no significant order effects were observed (see study limitation below). First, we compared the differences in temporal patterns of all variables between the high heat capacity gel mattress (HM) and the low heat capacity mattress (LM) conditions (Figures 1 and 2; Figures S1–S4). Next, we analyzed the relationships between sleep stages and all variables using nocturnal mean values for the entire night (Figure 3). Finally, we explored how the three physiological fields (sleep, body temperature, and cardiophysiology) might be interrelated by linear mixed effects model‐ (Figure 4) and mediation (Figure 5) analyses. Shortly summarized, the mattress‐induced increase of nocturnal mean values in lnHF is indirectly linked to the decline in CBT, mediated by increased IBI.

### 3.1 Analysis of nocturnal time courses

The analysis of the nocturnal time courses of body temperatures, IBI, and sleep stages has been previously published and will not be detailed here again (Herberger et al., 2020). Briefly, fast changes in all temperatures and cardiophysiological variables were observed in both mattress conditions (HM and LM) due to the effects of lying down and sleep initiation–PBT increases and CBT declines. These rapid changes lasted about 120 min and were superimposed by the slow cooling effect of the mattress, which led to a gradual reduction in PBT and CBT (Figure S1a,b). The minimum HM‐LM differences in the temperatures (Figure S2a,b) occurred approximately 150–180 min after lights off, when IBI reached its highest HM‐LM values (Figure 1a) (all MAT*TIME interactions, *p* < 0.0001; for statistics see Table S1). Significant differences between HM and LM are indicated by horizontal dotted lines in Figure S2a,b and Figure 2a (FDR, *p* < 0.05). In both mattress conditions, as well as in the HM‐LM differences, the nocturnal time course of lnHF (Figure 1b) and IBI (Figure 1a) showed similar patterns that were distinct from those of lnLF and LF/HF (Figure 1c,d), which were similar to each other.

Interestingly, the cardiophysiological variables (IBI, lnHF, lnLF, LF/HF) exhibit very fast changes directly after lights off. IBI and lnHF increased rapidly, and lnLF and LF/HF followed after a time lag of about 30 min. In all HRV measures, no statistically significant time course effects of mattress‐induced cooling (HM‐LM) were found; the MAT*TIME interaction did not reach statistical significance (Figures 1 and 2c–d; Table S1). Moreover, visual inspection of the data showed similar nocturnal time courses in the HM‐LM differences and for both mattress conditions, with lnHF and IBI following the same patterns as lnLF and LF/HF (Figures S3 and S4). Given the complexity of the temporal relationships between variables and time courses, a separate follow‐up analysis on this topic using cross‐correlation analysis is necessary and currently in progress. Nevertheless, we present an analysis between lnHF and IBI using mixed effects models for lnHF with factors IBI, MAT, and TIME (see below Table 1). IBI has been revealed as the most important variable to explain lnHF.

**TABLE 1:** Mixed effects model for lnHF with factors IBI, MAT, and TIME.

	numDF	denDF	*F*‐value	*p‐*value
Intercept	1	2714	12211.718	**< 0.0001**
IBI	1	2714	349.389	**< 0.0001**
MAT	1	30	1.482	0.2330
TIME	47	2714	1.043	0.3926
IBI*MAT	1	2714	0.493	0.4827
IBI*TIME	47	2714	0.559	0.9935
MAT*TIME	47	2714	0.959	0.5535
IBI*MAT*TIME	47	2714	0.688	0.9482

*Note*: Significant *p*‐values are bolded. The linear association between IBI and lnHF is not influenced by TIME and MAT, highlighting their close relationship.

Abbreviations: denDF, denominator degrees of freedom; IBI, factor Inter Beat Interval; lnHF, natural logarithm of the power in the High Frequency band (0.15–0.4 Hz); MAT, factor MAT (HM vs. LM); numDF, numerator degrees of freedom; TIME, factor TIME (48 × 10 min interval).

### 3.2 Nocturnal mean values of all measured variables across sleep stages

All measured variables revealed significant differences in nocturnal values between the sleep stages (Figure 3a–f, left panel), which were not affected by the mattress types (for statistics see Table S2 and Legend to Figure 3).

PBT was significantly lower during WAKE than NREM and REM (WAKE < NREM ≈ REM; FDR, *p* < 0.05; Figure 3a), and CBT tended to be lower during NREM than REM and WAKE (NREM < REM ≈ WAKE, *p* < 0.1; Figure 3b). IBI showed an inverse pattern to CBT, with the highest values during NREM and the lowest during WAKE (NREM > WAKE, *p* < 0.05; Figure 3c), with REM being intermediate. LnHF revealed the highest values during NREM, followed by WAKE, with the lowest values during REM (NREM > WAKE > REM, *p* < 0.05; Figure 3d), a pattern distinctly different from IBI. LnLF showed inverse patterns to LnHF, with the highest values during REM, the lowest during NREM, and intermediate values during WAKE (REM > WAKE > NREM; Figure 3e). The pattern of LF/HF was very similar to that of LnLF (Figure 3f), though with more pronounced statistical differences.

Compared to the LM (blue notched box‐and‐whisker plots), the HM (red notched box‐and‐whisker plots) significantly reduced 8 h‐mean nocturnal values of CBT and PBT, significantly increased IBI and lnHF, but did not affect lnLF and LF/HF (Figure 3a–f, upper graphs). The HM‐LM differences (∆) for all measured variables were not significantly modulated by sleep stages (Figure 3a–f, cooling effects of HM; for statistics see Table S2).

### 3.3 Effects of mattress induced temperature changes on cardiophysiological variables

A detailed analysis of the relationship between nocturnal mean values of mattress‐induced effects on IBI and lnHF showed significant correlations both intra‐individually (Figure 4, lower graphs) and inter‐individually (Figure 4, upper graphs). A mixed‐effects model further examined whether the relationship between IBI and lnHF is influenced by different nocturnal time courses (factor TIME) of the variables and/or by the different mattress conditions (factor MAT). The results in Table 1 indicate no significant statistical influence of these factors on the IBI and lnHF relationship [lnHF (ms^2^) = IBI (ms)*0.00203 (ms^2^ms^−1^) ± 0.00013, df = 2714, *t* = 16.0528, *p* < 0.0001] – all other factors did not significantly improve the prediction. This finding will be cited as an argument for a close relationship between IBI and lnHF.

In a final step, we explored how the cooling mattress‐induced changes might influence lnHF. A mediation analysis revealed that CBT cooling is not directly associated with increased lnHF (Figure 5). Instead, we identified a significant indirect pathway (*p* < 0.008): changes in CBT lead to changes in IBI, which then result in changes in lnHF. Therefore, ∆IBI is an significant mediator in this effect. When the same analysis was performed using ∆lnLF or ∆LF/HF as target variables, no significant mediating effect for ∆IBI was found (data not shown).

## 4 DISCUSSION

Our study primarily focused on examining whether and how gentle, non‐disruptive core body cooling affects HRV during sleep. Previous studies using the same cooling mattress type consistently found an increase in NREM sleep, particularly in N3 (Kräuchi et al., 2018; Reid et al., 2021; Herberger et al., 2020), which was also associated with an increase in IBI (Reid et al., 2021; Herberger et al., 2020). However, the relationship between the cooling effects of the mattress (HM) and changes in HRV was not analyzed in those studies, which we address in the present paper.

We have organized the data analysis of all measured variables into three main sections: (1) Time courses without considering sleep stages; (2) Comparison of nocturnal mean values across different sleep stages; and (3) Mattress‐induced temperature changes and their impact on cardio‐physiological variables. This paper does not address the relationship between NREM‐REM sleep cycles and the time courses of cardio‐physiological variables, which will be examined in a separate report.

### 4.1 Analysis of nocturnal time courses

In everyday life, sleep typically occurs at night when CBT is low. The onset of sleep usually coincides with the maximum decline in CBT in the evening (Campbell & Broughton, 1994), which is triggered by a decrease in body heat production and an increase in distal skin blood flow, leading to a greater body heat loss. In addition to these circadian changes, an additional reduction in CBT arises with sleep initiation (such as lying down, relaxation) that is primarily due to similar thermoregulatory effects, which may vary with age (Herberger et al., 2024) (Supplement). However, changes in sleep pressure (sleep propensity) at sleep onset, induced by factors such as sleep deprivation or satiation, do not significantly impact the thermoregulatory system (Dijk & Czeisler, 1993; Kräuchi et al., 2006), suggesting a complex relationship between these physiological systems. Our study design does not fully address these issues. Further research is needed to explore varying sleep times relative to the circadian CBT pattern and to modify sleep preparatory behaviors, such as through the use of appropriate forced desynchrony protocols across different age groups.

Assuming that both the slow circadian changes and the rapid physiological changes at sleep onset are similarly expressed across both mattress types, subtracting the LM values from the HM values should effectively eliminate these influences. This would isolate the ‘pure’ effects of mattress cooling on all physiological variables. Therefore, our interpretations primarily focus on these effects. In our study, the nocturnal time courses of CBT and, inversely, IBI were similarly modulated across both mattress types, including the effects of the cooling mattress (HM‐LM; see Figures S1 and S3; Table S1). These findings support the hypothesis that the HM‐induced reduction in CBT may directly influence IBI. It has long been known that changes in heat production result in corresponding changes in HR. This is based upon the linear relationship between HR and O_2_‐consumption except during very low and very high exercise intensity, making heart rate and hence IBI a reliable surrogate for estimating energy expenditure (Berggren & Hohwu, 1950; Kräuchi & Wirz‐Justice, 1994; Montgomery et al., 2009).

However, chronobiological studies have shown in constant routine protocols, circadian variations in HR are significantly associated with CBT, but with a phase advance of about 100 min (Kräuchi & Wirz‐Justice, 1994). In contrast, the circadian HR rhythm remains phase‐locked to O_2_‐consumption (heat production) (Kräuchi & Wirz‐Justice, 1994). The common explanation for this is that the circadian system, with the SCN as the master clock, controls the phase relationships between the rhythm of heat production and heat dissipation. The difference between them determines body heat content, and consequently, CBT (Aschoff, 1974). In contrast, acute physiological effects, such as those at sleep onset, are regulated by the thermoregulatory system and induce only small phase shifts between O_2_‐consumption, CBT, and heat dissipation (Kreider & Iampietro, 1959). Furthermore, HR is directly influenced by temperature. Sinus node activity is closely linked to temperature changes, even in vitro, and decreases as body temperature drops (Langer et al., 1999; Frey et al., 1996; Papaioannou et al., 2013). This effect occurs rapidly and does not induce a phase shift of IBI relative to CBT. It is therefore quite possible that the cooling mattress causes the increase in IBI through core body cooling (Herberger et al., 2024).

From a chronobiological point of view, the nocturnal decrease in CBT and the increase in IBI induced by the cooling mattress resemble a pattern similar to an increase in circadian amplitude. However, such a mechanism has not been described, and it is more likely a masking effect that does not directly influence the amplitude of the endogenous pacemaker, although the physiological effects may still be the same (Aschoff, 1983; Mrosovsky, 1999). Future studies are needed to investigate whether reduced CBT and increased IBI are associated with decreased nocturnal blood pressure (Besson et al., 2025; Shaffer & Ginsberg, 2017), which could have important therapeutic implications (presently under investigation).

Studies have shown that HRV measures, such as HF and LF, follow a distinct day‐night pattern, with lower HRV during the day and higher values during sleep (Glos et al., 2014; Mao et al., 2024). This pattern persists under constant routine and forced desynchrony conditions, which control for sleep and circadian phase, indicating that LF and HF have robust circadian rhythms, peaking during the circadian trough (Anders et al., 2010; Boudreau et al., 2012; Glos et al., 2014; Scheer et al., 2010; Vandewalle et al., 2007), typically coinciding with sleep. Given that the CBT rhythm reflects the activity of the circadian clock in both amplitude and phase, our findings could suggest a significant association of CBT with HRV. However, it remains unclear whether CBT and HRV are directly linked or only by their synchronous circadian patterns.

Immediately after lights off, we observed rapid changes in all measured cardiophysiological variables, along with thermoregulatory changes, such as an increase in PBT and a decrease in CBT. IBI and lnHF increased quickly, while lnLF showed a rise after a time lag of approximately 30 minutes, leading to a reduced LF/HF ratio (Figure S3b) (Anders et al., 2013; Bigalke et al., 2023; Trinder et al., 2001). The simultaneous rapid increase in IBI supports this notion, as parasympathetic activation slows down the HR (de Geus et al., 2019). Other studies have shown that sympathetic activity, such as that measured by the pre‐ejection period of the EEC, also decreases after lights off and sleep onset, which would lead to amplification of a reduced LF/HF ratio (Burgess et al., 1997).

### 4.2 Nocturnal mean values across sleep stages

The most striking finding from the sleep stage analysis is that mattress‐induced differences in nocturnal mean values for all measured variables did not significantly vary across sleep stages (Figure 3 black bars; no significant interaction MAT vs. STAGE, Table S2). This suggests that body cooling and its physiological effects occur independently of the sleep stages (including WAKE). Compared to LM, the cooling mattress HM reduced CBT and PBT, while IBI and lnHF increased; there was no change in lnLF or LF/HF with cooling. This finding is consistent with earlier studies, which suggest that parasympathetic nervous system activity, as indicated by HF, is primarily influenced by the slow‐changing circadian system, reflected by CBT. In contrast, sympathetic nervous system activity, as measured by the pre‐ejection period, is more strongly influenced by sleep (Burgess et al., 1997, 2001).

Based on these findings described above, the sleep stage analyses could be performed independently of mattress type (pooled data of HM and LM, see 8 hr‐sleep values in Figure 3). Significant differences in nocturnal means were observed between NREM and REM for all HRV measures (lnHF, lnLF, LF/HF), and tendentially for IBI, but not for CBT, PBT, as has been similarly described previously (Kräuchi & Deboer, 2013; Herberger et al., 2024). However, small mammals (e.g., mice, rats) show marked NREM‐REM sleep cycle‐modulated body temperature changes (Alföldi et al., 1990). It is therefore possible that the effects of the NREM‐REM cycles on body temperature in humans are less pronounced and thus harder to measure compared to small laboratory animals with a higher specific metabolic rate.

lnLF and LF/HF, when compared to lnHF, exhibit inverse patterns across sleep stages. Specifically, lnHF and LF/HF are highest during NREM sleep and lowest during REM sleep, while lnLF and LF/HF show the opposite pattern. This inverse relationship, also described by previous authors, has been attributed to the shift from parasympathetic dominance during NREM to sympathetic dominance during REM (Somers et al., 1993; Tobaldini et al., 2013; de Zambotti et al., 2013). Since the sleep‐stage pattern of IBI is not directly reflected by lnHF or lnLF, the relationship between IBI and these HRV measures appears more complex and is not simply a mirror image of IBI (Monfredi et al., 2014). However, these differences observed across the 8‐hour sleep phase may not be directly caused by the sleep stages themselves, but rather by their timing throughout the night. More specifically, early in the night, CBT decreases due to blood redistribution from the core to the periphery induced, for example, by lying down, leading to lower CBT and higher skin temperatures, including PBT (Krauchi, 2007). In addition to sleep‐preparatory behavioral thermoregulation, the endogenous circadian regulation of CBT exhibits similar thermoregulatory changes, particularly during the night when we usually sleep. These changes are associated with the appearance of NREM sleep, especially N3. Towards the end of the night, the opposite pattern occurs: REM and WAKE increase while NREM declines, coinciding with peripheral vasoconstriction, a rise in CBT, and culminating in wakefulness (Versace et al., 2003). Similarly, it can be argued that for all other variables, the sleep‐induced and circadian components overlap during sleep. This overlap can result in a nocturnal pattern where sleep states are unevenly distributed, leading to differences that are not solely induced by variations in sleep stages. To what extent this explanation holds true across the sleep stages remains to be determined.

### 4.3 Effects of mattress induced temperature changes on cardiophysiological variables

In the final analysis, we examined how a reduction in CBT does not have a direct effect on HF (Figure 5). We identified a significant indirect pathway: a reduction in CBT leads to increased IBI, which subsequently influences HF. Notably, when we conducted the same analyses using ∆lnLF or ∆LF/HF as target variables, we found no significant mediating effect of ∆IBI (data not shown), underscoring the specificity of this mediation effect.

It has frequently been noted that the mean and variability of IBI are highly correlated (Monfredi et al., 2014). Our study revealed heterogeneous patterns in lnLF, lnHF, and IBI across HM, LM, and HM‐LM conditions, influenced by factors such as the sleep initiation phase, nocturnal time course, and changes in sleep stages (see Figures 1 and 2, 3). This complexity makes a simple explanation unlikely, such as the idea that changes in HRV values are solely caused by changes in IBI values. Therefore, we do not support the notion that IBI is a surrogate for HRV, as it does not merely reflect redundancy that can be normalized by correction factors (Sacha, 2013). The intricate relationship between the mean and variability of the IBI highlights the need to report changes in HRV in relation to IBI dynamics (de Geus et al., 2019). Furthermore, our results are consistent with findings that CBT reduction leads to a higher tonic level of vagal activity and increases both HF and mean IBI (Walsh, 1969). Additionally, this heightened vagal activity could lead to an increased mean IBI by slowing the spontaneous diastolic depolarization of the sinoatrial pacemaker cells (de Geus et al., 2019). However, our study does not allow us to draw a reliable final conclusion about the mechanism of action.

Taken together, the most interesting finding of this study is that slow, conductive body heat loss during nocturnal sleep reduces PBT and CBT, increases IBI, and leads to distinct changes in HRV measures: lnHF increases, while lnLF and LF/HF remain unchanged. The well‐defined thermophysiological intervention allowed us to demonstrate a potential causal chain in which the selective increase in lnHF (an indicator of parasympathetic activation) results from CBT cooling mediated by IBI. In contrast, the modulation of HRV measures during sleep stages is independent of temperature effects (core body cooling) and not solely dependent on changes in IBI. The most plausible explanation for this finding is that the central efferences of the autonomic nervous system (sympathetic and parasympathetic activity) are modulated by the NREM–REM cycle (Chouchou & Desseilles, 2014).

### 4.4 Study limitation

Our study has several limitations that should be acknowledged. We did not include adaptation nights, as our primary focus was not on the effects of adaptation but on the impact of the mattress intervention itself. However, due to the randomized order of mattress types across study nights, no significant order effects were observed. This suggests that the observed mattress effects are reliable within the context of our study.

Another limitation is that the study included only male participants. As a result, the findings can, strictly speaking, only be generalized to the male population. To allow for broader conclusions, future studies should include mixed‐gender samples, ideally with a larger overall sample size.

Finally, we did not measure breathing physiology, although it is well established that respiratory parameters can strongly influence HRV. Both breathing frequency and tidal volume significantly affect HRV– especially the high‐frequency component associated with respiratory sinus arrhythmia, which is the result of cardiac‐respiratory interaction. Although the inclusion of respiratory data would have been valuable, tidal volume in particular is methodologically difficult to assess reliably during sleep and cannot be easily measured casually. For this reason, we chose not to monitor respiratory activity in this study. This should be taken into consideration in future studies focusing on respiratory physiology in the context of mild body cooling and HRV during sleep.

### 4.5 Practical implications

Since a decrease in HR during sleep is an important indicator of cardio‐restorative function (i.e., HR dipping), our findings suggest that mild body cooling during sleep may help enhance cardiovascular health. The middle‐aged men in this study were healthy and had no history of sleep disorders; therefore, greater effects may be observed in individuals with sleep disturbances (e.g., individuals with insomnia). Within this context, an important question arises: is the observed reduction in HR also associated with a decrease in nocturnal blood pressure? If so, this could have additional therapeutic implications– this possibility is currently under investigation. Furthermore, the findings of this study can serve as a basis for future research examining the potential role of mild body cooling in enhancing N3 sleep and its effects on cardiometabolic function, as well as cognitive outcomes in both healthy and clinical populations. Last but not least, in the context of climate‐related changes (e.g., rising temperatures), a cooling gel mattress could be a welcome aid for improving sleep by increasing conductive heat transfer from the body to the mattress.

## AUTHOR CONTRIBUTIONS

Conception and design of research: M.M, K.K, I.F., T.P. Investigation: T.P., I.F., S.H., M.G. Data analysis: M.G., K.K. Funding acquisition: M.M, T.P. Interpretation of the results: K.K, S.H. I.F., T.P. Writing of original draft and figures: K.K., S.H. Writing – review & editing: K.K., S.H. Approval of final manuscript: K.K., S.H.

## FUNDING INFORMATION

The study was financially supported by an unrestricted grant from Technogel Italia S.r.l., Vicenza, Italy. The authors alone are responsible for the content and writing of the manuscript.

## CONFLICT OF INTEREST STATEMENT

The authors report no conflicts of interest, either financial or of any other kind.

## Supporting information


Data S1.
**Table S1:** ANOVA of nocturnal time courses for all measured variables with factors MAT and TIME.
**Table S2:** ANOVA–table for nocturnal mean values per factors sleep STAGE and MAT.
Figures S1–S4.

## Data Availability

All data analyzed in this study is available upon reasonable request by inquiry to the corresponding authors Sebastian Herberger or Kurt Kräuchi. Requests for materials should be addressed to S.H. or K.K.
