# Supplementary material for: Slow nocturnal body cooling during sleep increases interbeat intervals and is tightly coupled to high‐frequency heart rate variability in healthy men
Source: Physiol Rep. 2025 Aug 7;13(15):e70478. doi: 10.14814/phy2.70478 (PMC12329345; doi:10.14814/phy2.70478)
Supplement: Supplementary file 1 — Data S1. Table S1: ANOVA of nocturnal time courses for all measured variables with factors MAT and TIME. Table S2: ANOVA–table for nocturnal mean values per factors sleep STAGE and MAT. Figures S1–S4. [file PHY2-13-e70478-s001.docx]

**Supplementary Material**

**Suppl. Table 1. ANOVA of nocturnal time courses for all measured variables with factors MAT and TIME**

**MAT TIME MAT*TIME**

Thermo. var.: F(1,29) p η^2^ F(47,1363) p η^2^ F(47,1363) p η^2^

**CBT** 5.760 **0.023** 0.040 50.474 **<0.0001** 0.273 3.365 **<0.0001** 0.020

**PBT** 8.396 **0.007** 0.038 19.762 **<0.0001** 0.174 6.990 **<0.0001** 0.048

Cardio. var.: F(1,29) p η^2^ F(47, 1363) p η^2^ F(47, 1363) p η^2^

**IBI** 10.194 **0.003** 0.021 2.133 **<0.0001** 0.008 2.136 **<0.0001** 0.005

**lnHF** 5.077 **0.032** 0.012 1.614  **0.0060** 0.011 1.204 0.165 0.007

**lnLF** 1.949 0.173 0.004 3.935 **<0.0001** 0.031 1.313 0.078 0.008

**LF/HF** 0.341 0.564 0.000 2.926 **<0.0001** 0.034 1.377 0.048 0.013

Sleep var.: F(1,30) p η^2^ F(47,1410) p η^2^ F(47,1410) p η^2^

**NREM** 4.877 **0.035** 0.002 9.002 **<0.0001** 0.129 1.329 0.069 0.019

**REM** 0.542 0.467 0.001 4.463 **<0.0001** 0.064 0.949 0.573 0.016

**WAKE** 4.666 **0.039** 0.004 12.297 **<0.0001** 0.171 1.082 0.329 0.013

**Legend to Suppl. Table1:**

Thermo. var.= thermophysiological variables: CBT=core body temperature, PBT=proximal back skin temperature; Cardio. var.= cardiophysiological variables: IBI=inter beat interval, lnHF=natural logarithm of the power in the high frequency band (0.15-0.4Hz), lnLF=natural logarithm of the power in the low frequency band (0.05-0.15Hz), LF/HF=coefficient of power in the low frequency band / power in the high frequency band; Sleep var.=sleep variables: NREM=non- rapid eye movement sleep (sleep stages N1+N2+N3), REM=rapid eye movement sleep. η^2^= generalized eta-squared measure of effect size (small η^2^ =0.01; medium η^2^ =0.06; large η^2^ =0.14; (21)). Significant p-values are bolded. N=31; F(numerator, denominator)= F(degrees of freedom). MAT = factor mattress (HM vs. LM), TIME = factor time course (48*10min interval)

**Suppl. Table 2. ANOVA -table for nocturnal mean values per factors sleep STAGE and MAT (N=31)**

**MAT STAGE MAT*STAGE**

Variable: F(1,30) p η^2^ F(2,60) p η^2^ F(2,60) p η^2^

**lnLF** 0.816 0.374 0.002 17.408 **<0.0001** 0.065 1.053 0.336 0.001

**lnHF** 4.272 **0.047** 0.013 35.879 **<0.0001** 0.085 1.044 0.358 0.001

**LF/HF** 1.091 0.305 0.003 25.850 **<0.0001** 0.162 0.946 0.348 0.002

**IBI** 8.014 **0.008** 0.018 21.014 **<0.0001** 0.033 1.111 0.336 0.001

**CBT** 7.201 **0.012** 0.018 18.499 **<0.0001** 0.026 0.156 0.856 0.002

**PBT** 7.921 **0.009** 0.059 19.932 **<0.0001** 0.085 1.892 0.161 0.003

**Legend to Suppl. Table2:**

See Legend to Suppl. Table1

STAGE = factor sleep stage, NREM, REM vs. WAKE

MAT = factor mattress, high heat capacity mattress (HM) vs. low heat capacity mattress (LM)

Similar results were found by non-parametric tests: MAT (HM vs LM; Wilcoxon-test), STAGE (NREM vs. REM vs W; Friedman-test), MAT*STAGE (HM-LM of NREM vs. HM-LM of REM vs. HM-LM of W; Friedman-test).

**Supplemental Figures 1-4**

Suppl.Fig.1 Suppl.Fig.2 Suppl.Fig.3 Suppl.Fig.4


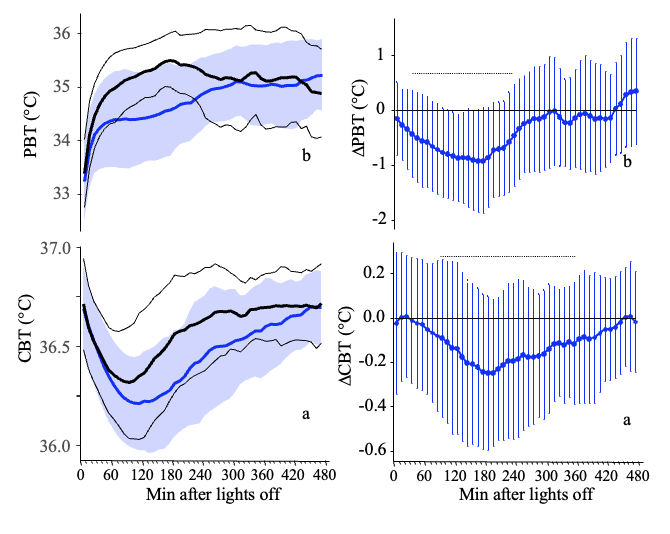

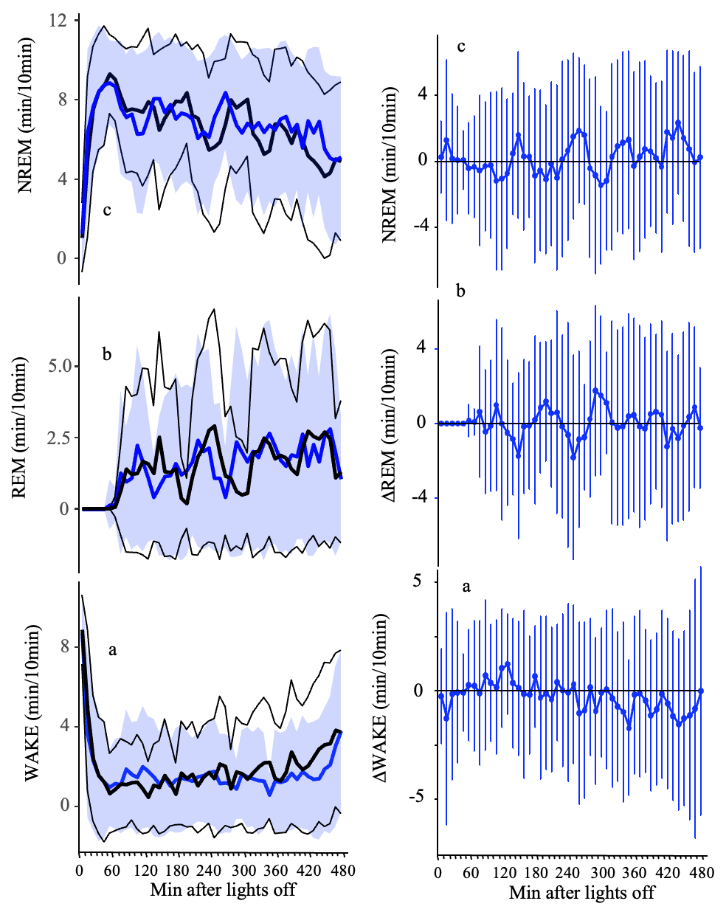


**Legend to Suppl. Figure 1&2:**

From bottom to top, the nocturnal time courses of HM (in blue) and LM (in black) are shown for CBT and PBT (Suppl. Fig.1a,b) and for the differences between HM and LM (∆) (Suppl. Fig.2a,b), [mean (thick lines) ±SD (blue ribbon or thin lines), N=31, 48x10min interval]. Black 0-lines indicate no difference between HM and LM. For ∆CBT and ∆PBT significant different values between HM and LM are indicated by a dotted horizontal line (FDR, p<0.05; Suppl. Fig.2a-b). CBT=core body temperature, PBT=proximal back skin temperature.

Note: CBT and PBT exhibit similar time courses in HM-LM differences (∆).

**Legend to Suppl. Figure 3&4:**

From bottom to top, the nocturnal time courses of HM (in blue) and LM (in black) are shown for WAKE, REM and NREM (Suppl. Fig.3a-c) and for the differences between HM and LM (∆) (Suppl. Fig.4a-c), [mean (thick lines) ±SD (blue ribbon or thin lines), N=31, 48x10min interval]. ]. Black 0-lines indicate no difference between HM and LM. NREM=non- rapid eye movement sleep (sleep stages N1+N2+N3), REM=rapid eye movement sleep.

Note: All sleep stages did not exhibit significant time courses in HM-LM differences (∆).
